# The development of PubMed search strategies for patient preferences for treatment outcomes

**DOI:** 10.1186/s12874-016-0192-5

**Published:** 2016-07-29

**Authors:** Ralph van Hoorn, Wietske Kievit, Andrew Booth, Kati Mozygemba, Kristin Bakke Lysdahl, Pietro Refolo, Dario Sacchini, Ansgar Gerhardus, Gert Jan van der Wilt, Marcia Tummers

**Affiliations:** 1 Radboud Institute for Health Sciences, Radboud University Medical Center, Nijmegen, The Netherlands; 2 Health Economics and Decision Science (HEDS), School of Health and Related Research (ScHARR), University of Sheffield Regent Court, Sheffield, United Kingdom; 3 Department of Health Services Research, Institute for Public Health and Nursing Research (IPP), University of Bremen, Bremen, Germany; 4 Centre for Medical Ethics, University of Oslo, Oslo, Norway; 5 Institute of Bioethics and Medical Humanities, “A. Gemelli” School of Medicine, Università Cattolica del Sacro Cuore, Rome, Italy; 6 Donders Institute for Brain, Cognition and Behaviour, Radboud University Medical Center, Nijmegen, The Netherlands; 7 Health Sciences Bremen, University of Bremen, Bremen, Germany

**Keywords:** Patient preferences, Treatment outcome, Evidence-based medicine*, Information storage and retrieval/methods*, MEDLINE

## Abstract

**Background:**

The importance of respecting patients’ preferences when making treatment decisions is increasingly recognized. Efficiently retrieving papers from the scientific literature reporting on the presence and nature of such preferences can help to achieve this goal. The objective of this study was to create a search filter for PubMed to help retrieve evidence on patient preferences for treatment outcomes.

**Methods:**

A total of 27 journals were hand-searched for articles on patient preferences for treatment outcomes published in 2011. Selected articles served as a reference set. To develop optimal search strategies to retrieve this set, all articles in the reference set were randomly split into a development and a validation set. MeSH-terms and keywords retrieved using PubReMiner were tested individually and as combinations in PubMed and evaluated for retrieval performance (e.g. sensitivity (Se) and specificity (Sp)).

**Results:**

Of 8238 articles, 22 were considered to report empirical evidence on patient preferences for specific treatment outcomes. The best search filters reached Se of 100 % [95 % CI 100-100] with Sp of 95 % [94–95 %] and Sp of 97 % [97–98 %] with 75 % Se [74–76 %]. In the validation set these queries reached values of Se of 90 % [89–91 %] with Sp 94 % [93–95 %] and Se of 80 % [79–81 %] with Sp of 97 % [96–96 %], respectively.

**Conclusions:**

Narrow and broad search queries were developed which can help in retrieving literature on patient preferences for treatment outcomes. Identifying such evidence may in turn enhance the incorporation of patient preferences in clinical decision making and health technology assessment.

**Electronic supplementary material:**

The online version of this article (doi:10.1186/s12874-016-0192-5) contains supplementary material, which is available to authorized users.

## Background

The importance of incorporating patients’ preferences in medical decision making is increasingly recognized. There is a growing consensus that they improve doctor-patient relationship and patients’ treatment adherence (compliance) and satisfaction [1–6]. Especially when a treatment decision depends on weighing uncertainties, risks, costs or adverse effects, the input from the patient in the decision process is crucial [7–11]. Patients’ preferences are usually described as a preference between one treatment or another, but such preferences are difficult to generalize as they are very context-dependent. Therefore, it would be more relevant to retrieve information on treatment outcomes which might explain such preferences, e.g. risks on adverse events, or specific outcomes such as functional status.

Patients can and do differ in their preferences for treatment outcomes, and knowledge of this can help clinicians to better support their patients [11–13]. Furthermore, researchers and policy makers may use this information to improve the assessment of treatments, for example in the context of health technology assessment (HTA) programs and/or healthcare prioritization strategies [14–16].

Searching for information on preferences for treatment outcomes in medical literature, for instance using PubMed, can be time-consuming [17, 18]. Making search strategies more specific, by for instance searching on methodology, may be problematic since patient preferences are, or could be, elicited in many ways, e.g. through interviews, focus groups, questionnaires or multi criteria decision analysis [18, 19]. Heterogeneity in methods used and reporting styles makes it more difficult to retrieve relevant literature.

The aim of this study was to develop a search filter, similar to PubMed’s Clinical Queries, with high retrieval performance to retrieve scientific papers reporting empirical evidence on patients’ preferences for treatment outcomes.

## Methods

Search filters were developed and validated in accordance with prevailing methods, such as those by Haynes et al. [20]. The process involves two steps: 1) a comprehensive set of search terms and combinations of terms was constructed, and 2) performance measures of these (sets of) search terms were determined by comparing the results with a set of manually identified papers (‘gold standard’ or reference set).

### Development of the set of relevant papers (reference set)

A set of relevant papers was constructed by hand-searching 27 journals on papers reporting empirical evidence on patient preferences for treatment outcomes. The list of journals was selected on the basis of expert opinion from the authors of this paper, experts in patient preferences and information specialists (see Table 2). Journals were selected on their likelihood of publishing relevant papers. The hand-search was limited to English publications in the year 2011 (this year was chosen in recognition that patient preferences are increasingly under investigation, but articles from later years may not all be properly MeSH-indexed [21]). Comments, news, editorials and study protocols were excluded.

In the first round of screening, the full list of articles was scanned based on title and abstract by two authors independently (RvH and MT, or RvH and WK). All articles selected in the first round were examined full text to determine whether they actually reported empirical data on patient preferences for treatment outcome. Studies were included that described preferences for treatment outcomes qualitatively or quantitatively, on individual or group level, regardless of the methods used. Studies that only described treatment preferences (i.e. preference for treatment A over B) for decision involvement or information, or preferences concerning diagnosis were not selected unless they also described preferences for specific outcomes (e.g. fatigue, pain). Studies that were based on proxy measures (e.g. asking doctors for patient preferences) were also excluded. Any disagreements were resolved by consensus with a third author. The final set of articles was designated as reference set and used to generate search terms and determine retrieval performance.

To allow for internal validation of the search queries, all articles were randomized (1:1) between a development set and a validation set using Microsoft Excel. This randomization process was done in such a way that each journal was equally represented in both sets and that the amount of relevant articles was balanced between the development set and the validation set.

### Search term generation

The subset of reference papers in the development set was submitted to PubReMiner [22]. PubReMiner is an online resource to which PubMed search queries can be submitted to produce a list and frequency counts for all keywords (subheadings, title-words etc.) and MeSH-terms associated with the articles in that query. The resulting list of keywords and MeSH-terms was used as basis to generate possible search filters. The keywords were used with and without the following fields: [tw] (text word), [tiab] (title/abstract), [majr] (MeSH major topic), [sh] (subheading) and [mh] (MeSH heading).

Each single search-term found by PubReMiner was tested individually to determine its sensitivity (Se), specificity (Sp), accuracy (Ac) and Number Needed to Read (NNR) (see Table 1). The Se is a measure of the proportion of relevant articles retrieved compared with all relevant articles. A search filter high in Se can be used when relevant literature is expected to be scarce or when the other filters do not return enough relevant literature. Specificity is a measure for the non-retrieval of non-relevant citations [23]. A search filter high in Sp may be used if the likely effect of missing relevant literature is not considered critical (e.g. given a large amount of relevant literature available). Accuracy is defined as the proportion of articles correctly handled by the search strategy [20], and the NNR is defined as the average number of articles one needs to screen to find one relevant article [24]. Filters high on Ac and low on NNR return few irrelevant papers while minimizing the number of missed relevant papers.

**Table 1:** Formulas for calculating the sensitivity, specificity, and Number Needed to Read (NNR)

	Relevant	Not relevant	Total
Identified	A (true positives, correct inclusion)	B (false positives, incorrect inclusion)	Total identified
Not identified	C (false negatives, incorrect exclusion)	D (true negatives, correct exclusion)	Total not identified
Total	A + C (total relevant hits)	B + D (total not relevant hits)	A + B + C + D (total database)

Sensitivity: A/(A + C); Specificity: D/(B + D); Accuracy: (A + D)/(A + B + C + D); NNR: 1/[A/(A + B)]

All search terms which yielded a Se > = 25 % and a Sp > = 75 % were considered of potential use. Single terms were combined using the OR-operator and the combined performance measures were determined. If a two-term search combination had a Sp > =75 %, a Se > =50 % and an Ac > =75 % it was considered for expansion with a third keyword. Combinations were expanded with additional keywords until no further increase in performance measures was observed without violating the performance thresholds. The queries of combinations of terms were tested in a program written in the programming language C++. Optimal combinations of search terms were made for each of the performance measures separately.

### Internal validation

The internal validity of the search strategies was determined by administering the search queries to the validation set and determining the performance measures. Validity was determined by comparing the Se and Sp of the test set with that of the validation set.

## Results

**Fig. 1:**
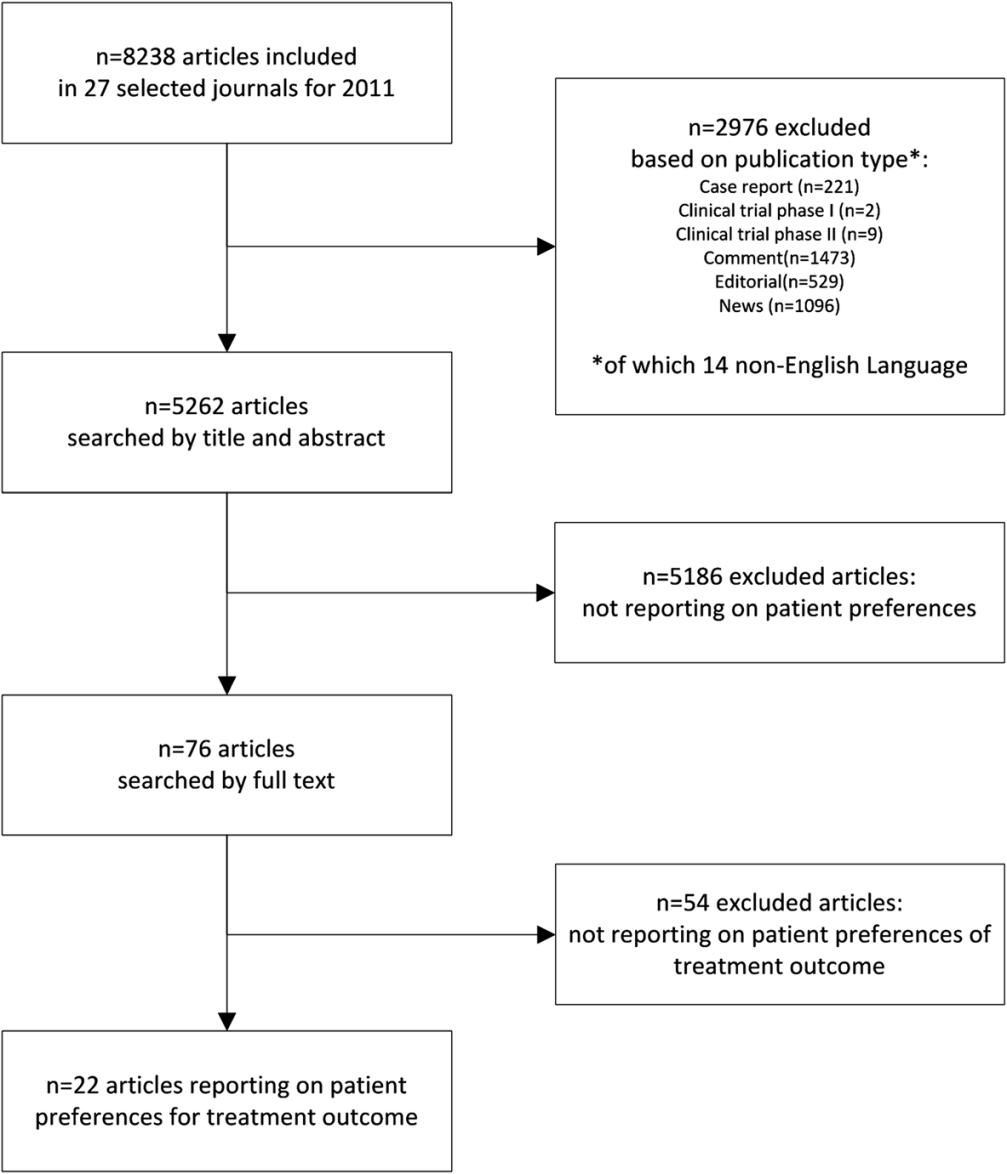
Manual search for relevant papers

A total of 8238 articles were screened on the basis of title and abstract. A total of 22 relevant articles (0.27 %) were selected with 100 % agreement as reference set (see Fig. 1). Table 2 lists the total number of articles in the development and test-set per journal. Additional file 1A contains a list of titles of the articles in the reference set.

**Table 2:** Number of articles in the validation set and the development set (total and number considered relevant) per journal

	Development set (relevant)	Validation set (relevant)	Total (relevant)
Annals of internal medicine	282 (0)	312 (0)	594 (0)
Anthropology and Medicine	9 (0)	16 (0)	25 (0)
Archives of internal medicine	269 (1)	245 (0)	514 (1)
BMC Health Services Research	182 (1)	190 (0)	372 (1)
BMC Medical Ethics	13 (0)	11 (0)	24 (0)
BMC Medical Informatics and Decision Making	34 (0)	43 (0)	77 (0)
BMC Medicine	80 (0)	59 (0)	139 (0)
British Medical Journal	1311 (0)	1293 (0)	2604 (0)
Culture, Medicine, and Psychiatry	19 (0)	8 (0)	27 (0)
Current medical research and opinion	123 (2)	147 (1)	270 (3)
Health Economics	54 (1)	63 (0)	117 (1)
Health Expectations	45 (0)	55 (1)	100 (1)
Implementation Science	68 (0)	65 (0)	133 (0)
International Journal of technology assessment in health care	28 (0)	34 (1)	62 (1)
Journal of general internal medicine	161 (0)	150 (1)	311 (1)
JAMA : the journal of the American Medical Association	504 (0)	503 (0)	1007 (0)
Journal of Clinical Epidemiology	99 (0)	98 (1)	197 (1)
Medical Anthropology Quarterly	11 (0)	15 (0)	26 (0)
Medical Care	86 (1)	90 (0)	176 (1)
Medical Decision Making	43 (0)	46 (1)	89 (1)
The patient	15 (1)	11 (1)	26 (2)
Patient Education and Counseling	182 (1)	196 (0)	378 (1)
Patient Preference and Adherence	32 (2)	37 (2)	69 (4)
Quality of life research	85 (0)	101 (0)	186 (0)
Social Science and Medicine	250 (1)	215 (0)	465 (1)
Sociology of Health and Illness	45 (0)	24 (0)	69 (0)
Value in Health	92 (1)	89 (1)	181 (2)
Total	4122 (12)	4116 (10)	8238 (22)

The papers were divided into a development set (*n* = 4122) which contained 12 papers from the reference set and a validation set (*n* = 4116) which contained 10 papers from the reference set. PubReMiner yielded a total of 162 MeSH-terms and 251 keywords from the 12 reference set papers in the development set, resulting in a total of 1668 single-term searches (a combination of each keyword with all search fields and the MeSH-terms) to be performed in PubMed. In these searches, 175 terms resulted in a Se > = 25 % and a Sp > = 75 %. Table 3 shows the top-three per performance measure of the single term searches. The best Se was found using Preferen*, reaching Se of 75 %, at the cost of Sp (97 %) and NNR (13.9). The best Sp, Ac and NNR could be gained with the keyword *Logit* (a term related to a specific type of regression model that is often used in discrete choice experiments which, in turn, are used to elicit patients’ preferences for treatment outcomes), yielding Sp and Ac > 99 % and an NNR of 2.8.

**Table 3:** Single term with the best sensitivity, best specificity, and lowest NNR for detecting articles reporting on patient preferences on treatment outcomes

Search term	Se (%) [95 % CI]	Sp (%) [95 % CI]	Ac (%)	NNR
Best sensitivity**				
Preferen*	75.0 [73.7–76.3]	97.2 [96.7–97.7]	97.1	13.9
Relat*[tiab]	75.0 [73.7–76.3]	79.5 [78.2–80.7]	79.4	94.8
“Middle Aged”[Mesh]	66.7 [65.2–68.1]	77.6 [76.3–78.9]	77.5	116.7
Best specificity**				
Logit	33.3 [31.9–34.8]	99.8 [99.7–100.0]	99.6	2.8
“Choice Behavior” [Mesh]	33.3 [31.9–34.8]	99.3 [99.0–99.6]	99.1	8.3
“Patient Preference” [Mesh]	50.0 [48.5–51.5]	99.3 [99.0–99.5]	99.1	6.0
Best accuracy**				
Logit	33.3 [31.9–34.8]	99.8 [99.7–100.0]	99.6	2.8
“Patient Preference”[mesh]	50.0 [48.5–51.5]	99.3 [99.0–99.5]	99.1	6.0
Choice Behavior[mh]	33.3 [31.9–34.8]	99.3 [99.0–99.6]	99.1	8.3
Lowest NNR**				
Logit	33.3 [31.9–34.8]	99.8 [99.7–100.0]	99.6	2.8
“Patient Preference”[mesh]	50.0 [48.5–51.5]	99.3 [99.0–99.5]	99.1	6.0
“Choice Behavior” [mesh]	33.3 [31.9–34.8]	99.3 [99.0–99.6]	99.1	8.3

*Se* Sensitivity, *Sp* Specificity, *Ac* Accuracy, *NNR* Number Needed to Read, *[tiab]* title/abstract, words and numbers included in the title, collection title, abstract, and other abstract of a citation, *[ti]* title, words and numbers included in the title or collection title. **Keeping sensitivity > =25 %, specificity > =75 %, and accuracy > = 75 %

Table 4 shows the best multi-term queries. Elicit* OR Choice* OR Prescrib*[tiab] yielded a Se of 100 % and a Sp of 95 %. It was possible to achieve a Se of 100 % with fewer terms, but at the expense of Sp. The top three search filters optimized for Sp, Ac and NNR were almost identical; for all three performance measures, “Patient Preference”[mesh] OR Preferen*[tiab] was the best combination (Se 75 %, Sp 97 %) to achieve high Ac or Sp, but a slightly lower NNR could be achieved with “Patient Preference”[mesh] OR Adheren*[tiab] (NNR was 13.2, and Se reached 83 %).

**Table 4:** Combinations of search terms with the best sensitivity, best specificity, and lowest NNR for detecting articles reporting on patient preferences on treatment outcomes (keeping sensitivity > 75 %, specificity > 50 %, and accuracy > 75 %)

Search term	Development set				Validation set			
	Se (%) [95 % CI]	Sp (%) [95 % CI]	Ac (%)	NNR	Se (%) [95 % CI]	Sp (%) [95 % CI]	Ac (%)	NNR
Best sensitivity								
Elicit* OR Choice* OR Prescrib*[tiab]	100.0 [100-100]	94.6 [93.9–95.3]	94.6	19.5	90.0 [89.1–90.9]	94.1 [93.3–94.8]	94.0	28.1
“Patient Preference”[mesh] OR Prescrib*[tiab] OR Elicit* OR Choice*[tiab]	100.0 [100-100]	94.3 [93.6–95.0]	94.3	20.6	80.0 [78.8–81.2]	93.8 [93.1–94.5]	93.8	32.9
“Patient Satisfaction”[mesh] OR Prescrib*[tiab] OR Logit OR Elicit*	100.0 [100-100]	94.0 [93.3–94.8]	94.1	21.4	80.0 [78.8–81.2]	94.4 [93.7–95.1]	94.3	29.9
Best specificity								
“Patient Preference”[mesh] OR Preferen*[tiab]	75.0 [73.7–76.3]	97.2 [96.7–97.7]	97.2	13.7	80.0 [78.8–81.2]	97.0 [96.4–97.5]	96.9	16.6
Preferen*	75.0 [73.7–76.3]	97.2 [96.7–97.7]	97.1	13.9	80.0 [78.8–81.2]	97.0 [96.4–97.5]	96.9	16.6
“Patient Preference”[mesh] OR Adheren*[tiab]	83.3 [82.2–84.5]	97.0 [96.5–97.6]	97.0	13.2	60.0 [58.5–61.5]	96.3 [95.8–96.9]	96.3	26.0
Best accuracy								
“Patient Preference”[mesh] OR Preferen*[tiab]	75.0 [73.7–76.3]	97.2 [96.7–97.7]	97.2	13.7	80.0 [78.8–81.2]	97.0 [96.4–97.5]	96.9	16.6
Preferen*	75.0 [73.7–76.3]	97.2 [96.7–97.7]	97.1	13.9	80.0 [78.8–81.2]	97.0 [96.4–97.5]	96.9	16.6
“Patient Preference”[mesh] OR Adheren*[tiab]	83.3 [82.2–84.5]	97.0 [96.5–97.6]	97.0	13.2	60.0 [58.5–61.5]	96.3 [95.8–96.9]	96.3	26.0
Lowest NNR								
“Patient Preference”[mesh] OR Adheren*[tiab]	83.3 [82.2–84.5]	97.0 [96.5–97.6]	97.0	13.2	80.0 [78.8–81.2]	97.0 [96.4–97.5]	96.9	16.6
“Patient Preference”[mesh] OR Preferen*[tiab]	75.0 [73.7–76.3]	97.2 [96.7–97.7]	97.2	13.7	60.0 [58.5–61.5]	96.3 [95.8–96.9]	96.3	26.0
Preferen*	75.0 [73.7–76.3]	97.2 [96.7–97.7]	97.1	13.9	80.0 [78.8–81.2]	97.0 [96.4–97.5]	96.9	16.6

*Se* Sensitivity, *Sp* Specificity, *Ac* Accuracy, *NNR* Number needed to read, *[tw]* text word field, *[sh]* MeSH subheading field, *[tiab]* title or abstract field, *[mesh]* MeSH term field

The internal validation results are shown in Table 4. There was a drop (of up to 19 %) in Se in the search terms optimized for sensitivity. There was no significant difference in Sp and the Ac also remained similar (a maximum drop of <1 %); the NNR increased to a maximum of 26 for the 12 presented filters. The three aforementioned best search queries, Elicit* OR Choice* OR Prescrib*[tiab], “Patient Preference”[mesh] OR Preferen*[tiab] and “Patient Preference”[mesh] OR Adheren*[tiab] yielded values of (Se 90 %, Sp 94 %), (Se 80 %, Sp 97 %) and (Se 60 %, Sp 96 %), respectively in the validation set.

## Discussion

Broad and narrow search filters were developed to allow for the efficient retrieval of scientific literature on patient preferences for treatment outcomes. The choice of filters may depend on the scope of the problem under investigation. A reasonable strategy might be to start with sensitivity-optimised filters, followed by specificity-optimised filters when the initial set of retrieved literature seems to vast and contaminated with marginally relevant papers. Clearly, the choice will also depend on the time-constraints and needs of the user.

The usefulness of these filters derives from the low prevalence of relevant studies in the scientific literature (less than 0.3 % in our manual search. Although currently no other search filters exist for retrieving literature on patient preferences for treatment outcomes, a comparison can be made between our search filters and the search filters underlying PubMed’s Clinical Queries (Haynes [25] and Wilczynski [26]). These search filters are used to find aetiology, prognosis, diagnosis or treatment related studies, targeting a variety of study types. Similar to the studies by Haynes and Wilczynski, our study produced many combinations of keywords reaching >99 % Se or Sp, but those performing good on one measure usually performed much worse on the other [25, 26]. However, where Haynes et al. reached an NNR of 1.7 - 4.8 (calculated from Table 7 in Haynes et al.), our filters reached an NNR of 13.2-21.4 (and even higher in the validation set). This difference might be explained by a significantly smaller set of papers deemed relevant in the reference set or a larger heterogeneity in the literature. Very low NNRs have been reported of search filters that have been developed to retrieve literature on specific disease conditions. For instance, the search filters for acute kidney injury content created by Hildebrand et al. reached NNRs of 1.2 [27]. This suggests that the difficulty of creating search filters for finding patients’ preferences for treatment outcomes might derive from the heterogeneity in context, type of study, intervention and outcomes [18, 19, 28].

The terms in the search filters appear to be largely associated with preferences and related keywords *between* treatments, not preferences towards treatment *outcomes*. This indicates that either preferences for treatment outcomes are difficult to distinguish from treatment preferences using search terms at an abstract level, or that a large heterogeneity in our set of relevant papers resulted in common terms to yield high enough performance measures.

The strength of our methodology is the testing of keywords without pre-selection and the validation of generated combinations of search terms in a separate set of papers.

A limitation of our study is the relatively low number of relevant papers that were found in the literature, increasing the odds of overfitting (i.e. making the filters too specific for our gold standard set) during the creation of the search filters. Due to the limited set of relevant papers, missing a single article will result in a drop of around 9 % in sensitivity, while specificity suffers much less due to its dependence on prevalence of relevant papers. Only 22 of all 8238 hand-searched articles (0.27 %) reported empirical evidence on patient preferences for treatment outcomes. There are two possible reasons for this finding: 1) there is little research performed on this subject, or the research is integrated into treatment preferences research; or 2) the research is inadequately reported at title and abstract level. If it is the latter, we may have missed these studies despite our thorough hand-searching methods. In either case, the shortage of articles implies that sensitive and comprehensive search strategies, like the ones described in this study, are essential for a successful literature search.

A second limitation of our study is that its focus is on general medical journals. Conceivably, slightly different terminology may be used in specific medical sub-specialties that could affect the performance of our search strings. In fact, when the literature source was extended to include the domain of Rheumatology (data not shown, but results are available in Additional file 1B), we found that search terms such as adheren* and choice* performed slightly better. Possibly, this reflects the relative importance of drug treatment in this area. For this reason, we suggest users determine whether the search filters identify key publications in the specific disease field.

Inevitably, the performance of the search strings presented in this paper reflects a particular terminology that was used by researchers who published findings of their work on patients’ preferences for treatment outcomes in 2011. It cannot be ruled out that certain changes take place in this terminology over time, which might affect the performance of the search strings presented in this paper. For this reason, an update of the performance of these search strings in a couple of years may be warranted. Alternatively, researchers in this area might be encouraged to employ the terminology that resulted in efficient retrieval of relevant papers. This, then, would likely further enhance the performance of these search strings in the future.

## Conclusion

Using standardized search methods for finding patient preferences for treatment outcomes may help clinicians, researchers and policy makers to understand patient preferences and further improve treatments or guidance. It may also help setting priorities or focus for further research (e.g. focus on decreasing the chance of a particular unpreferred outcome, instead of improving an entire treatment as a whole).

## Abbreviations

Ac, accuracy; NNR, number needed to read; Se, sensitivity; Sp, specify

## Additional file

Additional file 1: A contains the list of articles considered to contain relevant information (gold standard set). B contains the results from an analysis similar to the main article, except that for this analysis four additional Rheumatology journals are included in the set of abstracts. (PDF 3925 kb)
