# Supplementary material for: The development of PubMed search strategies for patient preferences for treatment outcomes
Source: BMC Med Res Methodol. 2016 Jul 29;16:88. doi: 10.1186/s12874-016-0192-5 (PMC4966584; doi:10.1186/s12874-016-0192-5)
Supplement: Additional file 1: — A contains the list of articles considered to contain relevant information (gold standard set). B contains the results from an analysis similar to the main article, except that for this analysis four additional Rheumatology journals are included in the set of abstracts. (PDF 3925 kb) [file 12874_2016_192_MOESM1_ESM.pdf]

Additional file 1 A
Articles identified as concerning 'patient preferences for treatment outcome' (n=22) during the manual search of the 27 journals for the year 2011 ('gold standard' subset)

Begum, S., Grunfeld, E. A., Ho-Asjoe, M., & Farhadi, J. (2011). An exploration of patient decision-making for autologous breast reconstructive surgery following a mastectomy. *Patient Educ Couns, 84*(1), 105-110. doi: 10.1016/j.pec.2010.07.004

Bogelund, M., Vilsboll, T., Faber, J., Henriksen, J. E., Gjesing, R. P., & Lammert, M. (2011). Patient preferences for diabetes management among people with type 2 diabetes in Denmark - a discrete choice experiment. *Curr Med Res Opin, 27*(11), 2175-2183. doi: 10.1185/03007995.2011.625404

Bonnichsen, O. (2011). Elicitation of ostomy pouch preferences: a discrete-choice experiment. *Patient, 4*(3), 163-175. doi: 10.2165/11586430-000000000-00000

Brinkman, W. B., & Epstein, J. N. (2011). Treatment planning for children with attention-deficit/hyperactivity disorder: treatment utilization and family preferences. *Patient Prefer Adherence, 5*, 45-56. doi: 10.2147/ppa.s10647

Danner, M., Hummel, J. M., Volz, F., van Manen, J. G., Wiegard, B., Dintsios, C. M., . . . Ijzerman, M. J. (2011). Integrating patients' views into health technology assessment: Analytic hierarchy process (AHP) as a method to elicit patient preferences. *Int J Technol Assess Health Care, 27*(4), 369-375. doi: 10.1017/s0266462311000523

Downar, J., Luk, T., Sibbald, R. W., Santini, T., Mikhael, J., Berman, H., & Hawryluck, L. (2011). Why do patients agree to a "Do not resuscitate" or "Full code" order? Perspectives of medical inpatients. *J Gen Intern Med, 26*(6), 582-587. doi: 10.1007/s11606-010-1616-2

Fiebig, D. G., Knox, S., Viney, R., Haas, M., & Street, D. J. (2011). Preferences for new and existing contraceptive products. *Health Econ, 20 Suppl 1*, 35-52. doi: 10.1002/hec.1686

Fried, T. R., Tinetti, M. E., Towle, V., O'Leary, J. R., & Iannone, L. (2011). Effects of benefits and harms on older persons' willingness to take medication for primary cardiovascular prevention. *Arch Intern Med, 171*(10), 923-928. doi: 10.1001/archinternmed.2011.32

Guo, N., Marra, C. A., FitzGerald, J. M., Elwood, R. K., Anis, A. H., & Marra, F. (2011). Patient preference for latent tuberculosis infection preventive treatment: a discrete choice experiment. *Value Health, 14*(6), 937-943. doi: 10.1016/j.jval.2011.05.003

Hauber, A. B., Gonzalez, J. M., Coombs, J., Sirulnik, A., Palacios, D., & Scherzer, N. (2011). Patient preferences for reducing toxicities of treatments for gastrointestinal stromal tumor (GIST). *Patient Prefer Adherence, 5*, 307-314. doi: 10.2147/ppa.s20445

Jewell, E. L., Smrtka, M., Broadwater, G., Valea, F., Davis, D. M., Nolte, K. C., . . . Havrilesky, L. J. (2011). Utility scores and treatment preferences for clinical early-stage cervical cancer. *Value Health, 14*(4), 582-586. doi: 10.1016/j.jval.2010.11.017

McCaffery, K. J., Turner, R., Macaskill, P., Walter, S. D., Chan, S. F., & Irwig, L. (2011). Determining the impact of informed choice: separating treatment effects from the effects of choice and selection in randomized trials. *Med Decis Making, 31*(2), 229-236. doi: 10.1177/0272989x10379919

Muller-Schwefe, G. H. (2011). European survey of chronic pain patients: results for Germany. *Curr Med Res Opin, 27*(11), 2099-2106. doi: 10.1185/03007995.2011.621935

Murad, M. H., Shah, N. D., Van Houten, H. K., Ziegenfuss, J. Y., Deming, J. R., Beebe, T. J., . . . Montori, V. M. (2011). Individuals with diabetes preferred that future trials use patient-important outcomes and provide pragmatic inferences. *J Clin Epidemiol, 64*(7), 743-748. doi: 10.1016/j.jclinepi.2010.08.005

Ross, S. A., Tildesley, H. D., & Ashkenas, J. (2011). Barriers to effective insulin treatment: the persistence of poor glycemic control in type 2 diabetes. *Curr Med Res Opin, 27 Suppl 3*, 13-20. doi: 10.1185/03007995.2011.621416

Schousboe, J. T., Davison, M. L., Dowd, B., Thiede Call, K., Johnson, P., & Kane, R. L. (2011). Predictors of patients' perceived need for medication to prevent fracture. *Med Care, 49*(3), 273-280. doi: 10.1097/MLR.0b013e318202915e

Sivell, S., Edwards, A., Elwyn, G., & Manstead, A. S. (2011). Understanding surgery choices for breast cancer: how might the Theory of Planned Behaviour and the Common Sense Model contribute to decision support interventions? *Health Expect, 14 Suppl 1*, 6-19. doi: 10.1111/j.1369-7625.2009.00558.x

Sweeting, K. R., Whitty, J. A., Scuffham, P. A., & Yelland, M. J. (2011). Patient preferences for treatment of achilles tendon pain: results from a discrete-choice experiment. *Patient, 4*(1), 45-54. doi: 10.2165/11532830-000000000-00000

Van Brunt, K., Matza, L. S., Classi, P. M., & Johnston, J. A. (2011). Preferences related to attention-deficit/hyperactivity disorder and its treatment. *Patient Prefer Adherence, 5*, 33-43. doi: 10.2147/ppa.s6389

Williams, B., Steven, K., & Sullivan, F. M. (2011). Tacit and transitionary: an exploration of patients' and primary care health professionals' goals in relation to asthma. *Soc Sci Med, 72*(8), 1359-1366. doi: 10.1016/j.socscimed.2011.02.038

Wirostko, B., Beusterien, K., Grinspan, J., Ciulla, T., Gonder, J., Barsdorf, A., & Pleil, A. (2011). Patient preferences in the treatment of diabetic retinopathy. *Patient Prefer Adherence, 5*, 229-237. doi: 10.2147/ppa.s11972

Zwijnenberg, N. C., Damman, O. C., Spreeuwenberg, P., Hendriks, M., & Rademakers, J. J. (2011). Different patient subgroup, different ranking? Which quality indicators do patients find important when choosing a hospital for hip- or knee arthroplasty? *BMC Health Serv Res, 11*, 299. doi: 10.1186/1472-6963-11-299

B

The results below are from an analysis similar to the main article, except that for this analysis four additional Rheumatology journals are included in the set of abstracts. This resulted in the following additional abstracts included in the analysis (table 1):

Table 1: Additional abstracts in the analysis

	**Development set (relevant)**	**Validation set (relevant)**	**Total (relevant)**
Annals of the Rheumatic Diseases	200 (0)	240 (2)	440 (2)
Arthritis care & research	144 (2)	134 (0)	278 (2)
Arthritis research & therapy	162 (0)	152 (0)	314 (0)
Arthritis and rheumatism	273 (0)	253 (0)	526 (0)
**Total**	**779 (2)**	**779 (2)**	**1558 (4)**

Four additional papers were identified that were considered relevant:

Bodar, E. J., Kuijk, L. M., Drenth, J. P., van der Meer, J. W., Simon, A., & Frenkel, J. (2011). On- demand anakinra treatment is effective in mevalonate kinase deficiency. Ann Rheum Dis, 70(12), 2155-2158. doi: 10.1136/ard.2011.149922

Heiberg, T., Lie, E., van der Heijde, D., & Kvien, T. K. (2011). Sleep problems are of higher priority for improvement for patients with ankylosing spondylitis than for patients with other inflammatory arthropathies. Ann Rheum Dis, 70(5), 872-873. doi: 10.1136/ard.2010.133793

Mann, C., & Gooberman-Hill, R. (2011). Health care provision for osteoarthritis: concordance between what patients would like and what health professionals think they should have. Arthritis Care Res (Hoboken), 63(7), 963-972. doi: 10.1002/acr.20459

van Hulst, L. T., Kievit, W., van Bommel, R., van Riel, P. L., & Fraenkel, L. (2011). Rheumatoid arthritis patients and rheumatologists approach the decision to escalate care differently: results of a maximum difference scaling experiment. Arthritis Care Res (Hoboken), 63(10), 1407-1414. doi: 10.1002/acr.20551

Table 2 and 3 show the results of the methods to create optimal filters using both single terms and combinations thereof including the abstracts mentioned above.

Table 2. Single term with the best sensitivity, best specificity, and lowest NNR for detecting articles reporting on patient preferences on treatment outcomes.

Search term	Se (%) [95% CI]	Sp (%) [95% CI]	Ac (%)	NNR
**Best sensitivity***				
"Delivery of Health Care"[mesh]	78.6 [77.4 - 79.7]	76.7 [75.6 - 77.9]	76.7	104.4
Healthcare[mh]	78.6 [77.4 - 79.7]	76.7 [75.6 - 77.9]	76.7	104.4
Relat*[tiab]	71.4 [70.2 - 72.7]	79.0 [77.8 - 80.1]	79.0	103.7
**Best specificity***				
Logit	28.6 [27.3 - 29.8]	99.8 [99.7 - 99.9]	99.6	3.0
"Patient Preference"[mesh]	42.9 [41.5 - 44.2]	99.4 [99.2 - 99.6]	99.3	5.7
Preferen*[ti]	42.9 [41.5 - 44.2]	99.4 [99.2 - 99.6]	99.3	5.7
**Best accuracy***				
Logit	28.6 [27.3 - 29.8]	99.8 [99.7 - 99.9]	99.6	3.0
"Patient Preference"[mesh]	42.9 [41.5 - 44.2]	99.4 [99.2 - 99.6]	99.3	5.7
Preferen*[ti]	42.9 [41.5 - 44.2]	99.4 [99.2 - 99.6]	99.3	5.7
**Lowest NNR***				
Logit	28.6 [27.3 - 29.8]	99.8 [99.7 - 99.9]	99.6	3.0
"Patient Preference"[mesh]	42.9 [41.5 - 44.2]	99.4 [99.2 - 99.6]	99.3	5.7
Preferen*[ti]	42.9 [41.5 - 44.2]	99.4 [99.2 - 99.6]	99.3	5.7

Se: Sensitivity, Sp: Specificity, Ac: Accuracy, NNR: Number Needed to Read, [tiab] = title/abstract, words and numbers included in the title, collection title, abstract, and other abstract of a citation, [ti] = title, words and numbers included in the title or collection title.
*Keeping sensitivity >=25%, specificity >=75%, and accuracy >= 75%.

Table 3. Combinations of search terms with the best sensitivity, best specificity, and lowest NNR for detecting articles reporting on patient preferences on treatment outcomes (keeping sensitivity > 75%, specificity > 50%, and accuracy > 75%)

Search term	Development set				Validation set			
**Best sensitivity**	**Se (%) [95% CI]**	**Sp (%) [95% CI]**	**Ac (%)**	**NNR**	**Se (%) [95% CI]**	**Sp (%) [95% CI]**	**Ac (%)**	**NNR**
"Patient Satisfaction"[mesh] OR prescrib*[tiab] OR experiment*[tiab] OR preferen*[ti]	100.0 [100 - 100]	93.8 [93.2 - 94.5]	93.8	22.5	75.0 [73.8 - 76.2]	93.3 [92.6 - 94.0]	93.3	37.1
choice* OR practitioner*[tiab] OR preferen*[ti] OR prescrib*[tiab]	100.0 [100 - 100]	93.8 [93.1 - 94.5]	93.8	22.6	83.3 [82.3 - 84.4]	93.6 [92.9 - 94..3]	93.5	32.6
"Patient Satisfaction"[mesh] OR ask*[tiab] OR preferen*[ti] OR prescrib*[tiab]	100.0 [100 - 100]	93.6 [92.9 - 94.2]	93.8	23.5	75.0 [73.8 - 76.2]	94.0 [93.3 - 94.7]	94.0	33.3
**Best specificity**								
"Patient Preference"[mesh] OR adheren*[tw]	78.6 [77.4 - 79.7]	96.3 [95.8 - 96.9]	96.3	17.3	58.3 [57.0 - 59.7]	95.7 [95.2 - 96.3]	95.6	31.0
choice* OR practitioner*[tiab]	78.6 [77.4 - 79.7]	96.0 [95.5 - 96.6]	96.0	18.6	58.3 [57.0 - 59.7]	95.6 [95.0 - 96.2]	95.5	31.7
"Patient Satisfaction"[mesh] OR preferen*[tiab]	78.6 [77.4 - 79.7]	95.9 [95.4 - 96.5]	95.9	19.0	75.0 [73.8 - 76.2]	96.0 [95.4 - 96.5]	95.9	23.0
**Best accuracy**								
"Patient Preference"[mesh] OR adheren*[tw]	78.6 [77.4 - 79.7]	96.3 [95.8 - 96.9]	96.3	17.3	58.3 [57.0 - 59.7]	95.7 [95.2 - 96.3]	95.6	31.0
choice* OR practitioner*[tiab]	78.6 [77.4 - 79.7]	96.0 [95.5 - 96.6]	96.0	18.6	58.3 [57.0 - 59.7]	95.6 [95.0 - 96.2]	95.5	31.7
"Patient Satisfaction"[mesh] OR preferen*[tiab]	78.6 [77.4 - 79.7]	95.9 [95.4 - 96.5]	95.9	19.0	75.0 [73.8 - 76.2]	96.0 [95.4 - 96.5]	95.9	23.0
**Lowest NNR**								
"Patient Preference"[mesh] OR adheren*[tw]	78.6 [77.4 - 79.7]	96.3 [95.8 - 96.9]	96.3	17.3	58.3 [57.0 - 59.7]	95.7 [95.2 - 96.3]	95.6	31.0
"Patient Satisfaction"[mesh] OR prescrib*[tiab] OR preferen*[ti]	92.9 [92.1 - 93.6]	95.3 [94.7 - 95.9]	95.3	18.7	75.0 [73.8 - 76.2]	95.4 [94.8 - 96.0]	95.4	25.7
Choice* or practitioner*[tiab]	78.6 [77.4 - 79.7]	96.0 [95.5 - 96.6]	96.0	18.6	58.3 [57.0 - 59.7]	95.6 [95.0 - 96.2]	95.5	31.7

Se: Sensitivity, Sp: Specificity, Ac: Accuracy, NNR: Number needed to read, [tw]: text word field, [sh]: MeSH subheading field, [tiab]: title or abstract field, [mesh]: MeSH term field
